# Dislocation of the Femur of 39 Days Standing Reduced by Manipulation

**Published:** 1860-01

**Authors:** 


### SURGICAL NOTES.

1. Dislocation of the Femur of Thirty-nine Days' Standing, Reduced by Manipulation.—The patient in this case was one of the wounded at the accident on the Chicago and North-Western Railway. The nature of the accident seems to have been misapprehended, and it had been treated as a fracture. The reduction by manipulation after this length of time presents many difficulties arising from the necessity of breaking up adhesions, and from the opening in the capsular ligament through which the head of the femur escapes from the socket being closed. By care and perseverance this was overcome, and the reduction effected. This is the third case already reported in these notes within a few weeks, in which this form of dislocation had been reduced. In only one was this effected by simply following the directions of Ried. In the other two, different movements were required to replace the bone in the acetabulum.

Much less pain and inflammation resulted from the efforts at reduction than would be expected after such a length of time, and ten days after the reduction, I learned that he was doing well, free from pain, with the member in the natural position.

